# Rehabilitation of persistent aphagia after Wallenberg syndrome by a novel combination method

**Published:** 2019-04-04

**Authors:** Payam Sariaslani, Dalir Parsa, Hiwa Mohammadi

**Affiliations:** 1 Department of Neurology, School of Medicine, Kermanshah University of Medical Sciences, Kermanshah, Iran; 2 Clinical Research Promotion Center, Imam Reza Hospital, Kermanshah University of Medical Sciences, Kermanshah, Iran; 3 Sleep Disorders Research Center, Kermanshah University of Medical Sciences, Kermanshah, Iran

**Keywords:** Wallenberg Syndrome, Dysphagia, Transcranial Direct Current Stimulation, Swallowing Rehabilitation

Lateral medullary syndrome (LMS) results from an occlusion of posterior inferior cerebellar or the vertebral artery. Clinical presentation may include dizziness, dysphagia, dysarthria, dysphonia, and ipsilateral face and contralateral body sensory deficits. Dysphagia is a common symptom with 51% prevalence among all patients with LMS, that accompanied by ipsilateral IX and X cranial nerve paralysis and diminished gag reflex resulted from nucleus ambiguus lesion.^1^ This group of large motor neurons located laterally in the upper medulla innervates the laryngeal and pharyngeal muscles. It is a part of a central pattern generator for swallowing function that innervates by corticobulbar upper motor neuron directly.^2^ Due to more cortical compared to subcortical plasticity, more considerable recovery of dysphagia has been reported when resulted from cortical lesion in comparison to brain stem accidents such as LMS. The last has longer duration or may be persistent.^3^

For oropharyngeal dysphagia, speech-language therapy and gastrostomy has been recommended, but the first has been ignored in many countries including Iran. Previous studies reported significant improvement of dysphagia using non-invasive brain stimulation.^4^ Data about the effects of these new therapeutic methods on LMS-induced chronic persistent aphagia is rare.

A 57-year-old man was hospitalized with acute onset vertigo, left paresthesia, and swallowing inability. On examination, unilateral right limb hyposensation for tactile, and left limb hyposensation for pain and heat were detected. Sensation and motor functions of face was bilaterally normal. Paralysis of the right palatoglossal and palatopharyngeal folds and impaired gag reflex were detected. Due to inability to swallow anything an orogastric tube was fixed. 

Magnetic resonance angiography (MRA), computed tomographic (CT) angiography, and color Doppler sonography failed to identify any arterial dissection or significant carotid and vertebral stenosis.

Brain MRI in T2WI and FLAIR revealed unilateral hypersignal lesion in the right medulla (Figure 1).

**Figure 1:**
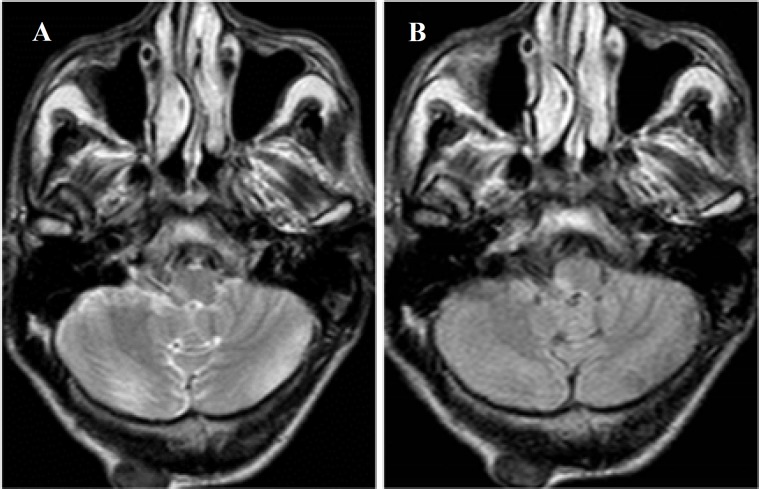
Brain magnetic resonance angiography (MRA) in T2WI (A) and FLAIR (B) revealed hypersignal lesion in the right medulla.

The Wallenberg's lateral medullary syndrome was diagnosed, and intervention has been started on subcutaneous low-molecular-weight heparin, dual antiplatelet therapy (aspirin and Plavix) and atorvastatin. After discharging from hospital, on 3 follow-up with one month intervals, his bilateral sensory deficits were recovered but his swallowing problem had not improved, and gradually weight loss was detected; so, gastrostomy was recommended.

Before gastrostomy, he was referred to speech-language pathology service. Moderately hyponasal speech, breathy voice, and slow laryngeal elevation during dry swallowing were detected. Videostroboscopy examination revealed unilateral abductor's paralysis of the right vocal and aryepiglottic folds (Figure 2). 

**Figure 2:**
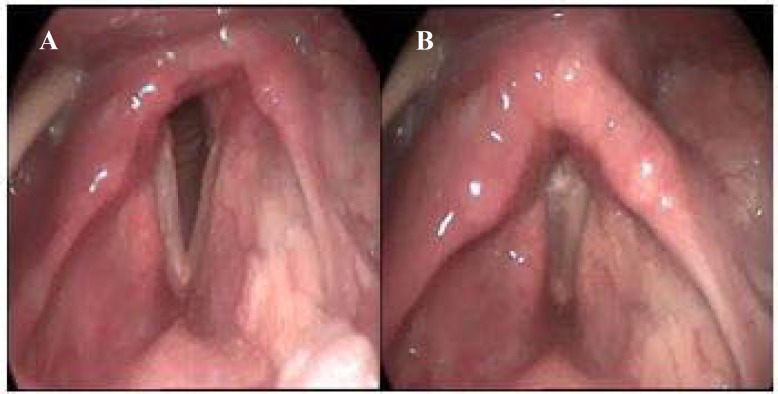
Top view of the larynx during inhalation (A) and phonation (B) documented by videostroboscopy three months after stroke and in the first visit of speech-language pathologist

Combination of speech therapy techniques and transcortical direct current stimulation (tDCS) were planned. Oromotor and respiratory exercises, supraglottic and Masako maneuvers, chin-tuck with effortful swallow, and shaker’s exercise were worked and taught to the caregivers for in-home practices. Simultaneously anodal tDCSs were presented to the right primary motor areas responsible for larynx and pharynx with intensity of 2 mA for 20 minutes. After session six, patient swallowed 5 cc water effortfully with subsequent wet voice and throat clearing. After session eight, partial oral diets with postural modification were started. After session 10, the videolaryngoscopy examination showed considerable motor recovery of the laryngeal unilateral paralysis (Figure 3). The OG tube was discontinued, and feeding with liquids and soft diets was started. Three follow-up examinations with 7 days intervals indicated more improvement in swallowing, and full oral diet was started. After a one month follow up, his weight was gradually increased.

**Figure 3:**
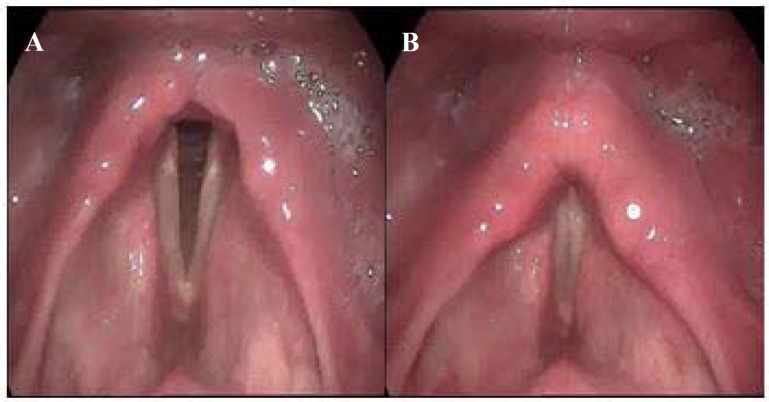
Videostroboscopy during inhalation (A) and phonation (B) after 10 session of treatment

In this case report, we presented a patients suffering from chronic aphagia following LMS, who gained his swallowing ability using an extensive speech-language pathology program combined with a tDCS regimen. Due to lack of in-patient swallowing rehabilitation and ignoring the speech-language pathology service, the aphagia was persistent for three months, and the patient was candidate for gastrostomy. Rejection of the gastrostomy by the patient led to referring him to swallowing rehabilitation service. 

Due to smaller amount of neuroplasticity in the brain stem, dysphagia conducted by LMS takes longer to recovery or even persist, and may lead to gastrostomy.^3^ Noninvasive brain stimulation methods such as transcranial magnetic stimulation (TMS) and tDCS has been investigated for dysphagia after cortical lesions, but data about their efficacy on LMS induced dysphagia are limited to TMS.^5^ Extensive speech-language pathology program combined with simultaneous anodal tDCS to ipsilateral oropharyngeal and laryngeal primary motor cortex led to complete swallowing ability and recovery of ipsilateral larynx and pharynx paralysis in our patient. Anodal tDCS can enhance motor functions by increasing corticomotor excitability.^4^ Combination of lower and upper motor stimulation by sensorimotor excitation and tDSC led to complete recovery of chronic aphagia in our case. According to the Hebbian theory, simultaneous stimulation of presynaptic and postsynaptic neurons increases synaptic efficacy.^6^^,^^7^ The present procedure may enhance neural repairing by simultaneously excitation of upper and lower motor neurons. 

Accumulation effects of motor exercises and anodal tDCS may resolve persistent aphagia after LMS. This combination procedure enhances neural reorganization of swallowing central pattern generator by simultaneously lower and upper motor excitation. This method should be evaluated in future studies.

## Notes:

**How to cite this article:** Sariaslani P, Parsa D, Mohammadi H. Rehabilitation of persistent aphagia after Wallenberg syndrome by a novel combination method. Iran J Neurol 2019; 18(2): 90-2.
